# Editors’ statement on the responsible use of generative AI technologies in scholarly journal publishing

**DOI:** 10.1007/s11019-023-10176-6

**Published:** 2023-10-20

**Authors:** Gregory E. Kaebnick, David Christopher Magnus, Audiey Kao, Mohammad Hosseini, David Resnik, Veljko Dubljević, Christy Rentmeester, Bert Gordijn, Mark J. Cherry

**Affiliations:** 1 The Hastings Center, Putnam Country, USA; 2 Stanford University, Stanford, USA; 3 American Medical Association, Chicago, USA; 4 Northwestern University, Evanston, USA; 5 U.S. National Institute of Environmental Health Sciences, Durham, USA; 6 North Carolina State University, Raleigh, USA; 7 Dublin City University, Dublin, Ireland; 8 St. Edwards University, Austin, USA

**Keywords:** Generative AI, Chatbots, Bioethics

## Abstract

Generative artificial intelligence (AI) has the potential to transform many aspects of scholarly publishing. Authors, peer reviewers, and editors might use AI in a variety of ways, and those uses might augment their existing work or might instead be intended to replace it. We are editors of bioethics and humanities journals who have been contemplating the implications of this ongoing transformation. We believe that generative AI may pose a threat to the goals that animate our work but could also be valuable for achieving those goals. In the interests of fostering a wider conversation about how generative AI may be used, we have developed a preliminary set of recommendations for its use in scholarly publishing. We hope that the recommendations and rationales set out here will help the scholarly community navigate toward a deeper understanding of the strengths, limits, and challenges of AI for responsible scholarly work.

## Introduction

The new generative artificial intelligence (AI) tools, and especially the large language models (LLMs) of which ChatGPT is the most prominent example, have the potential to transform many aspects of scholarly publishing. How the transformations will play out remains to be seen, both because the different parties involved in the production and publication of scholarly work are still learning about these tools and because the tools themselves are still in development, but the tools have a vast range of potential uses. Authors are likely to use generative AI to conduct research, frame their thoughts, produce data, search for ways of articulating their thoughts, develop drafts, generate text, revise their writing, and create visuals. Peer reviewers might use AI to help them produce their reviews. Editors might use AI in the initial editorial screening of manuscripts, to locate reviewers, or for copyediting.

We are editors of bioethics and humanities journals who have been contemplating the implications of this ongoing transformation. We believe that generative AI may pose a threat to the goals that animate our work but could also be valuable for achieving those goals. We do not pretend to have resolved the many social questions that we think generative AI raises for scholarly publishing, but in the interest of fostering a wider conversation about these questions, we have developed a preliminary set of recommendations about generative AI in scholarly publishing. We hope that the recommendations and rationales set out here will help the scholarly community navigate toward a deeper understanding of the strengths, limits, and challenges of AI for responsible scholarly work.

## Recommendations

### LLMs or other generative AI tools should not be listed as authors on papers

The argument usually given for prohibiting a generative AI tool from being listed as an author is that a requirement of morally responsible publishing is that authors must be accountable for what they write, and generative AI tools lack accountability (Hosseini et al. 2023a, b; International Committee of Medical Journal Editors 2023; Liebrenz et al. 2023; Lund et al. 2023; Teixeira and Tsigaris 2023). The publishing industry seems to have reached a consensus that this is a new norm for publishing, which creates a strong presumption in favor of acceptance. While arguments can be made that generative AI possesses some aspects of authorial accountability, such as the capacity to provide an account or explanation of how an article was created, the aspect of accountability that generative AI genuinely lacks is moral responsibility (Hosseini et al. 2023b). Only persons can be morally responsible, and therefore, if authors must possess moral responsibility, then generative AI cannot be an author.

This argument may seem to resolve the question of authorship by sheer stipulation: it accepts in principle that generative AI might (at least eventually) be able to generate content as well as human authors can, and it denies them authorial status by simply asserting that that role is reserved for full members of the moral community. The rationale for this stipulation is that it’s required to fulfill our journals’ mission. The goal of our journals is, in part, to foster a community of persons engaged in responsible thinking about ethical and social issues in health care and the biological sciences, not merely to generate publishable papers on those topics. The requirement for accountability is thus grounded both in an understanding of morally responsible publishing and in a goal of creating and protecting a community of people engaged in our work.

### Authors should be transparent about their use of generative AI, and editors should have access to tools and strategies for ensuring authors’ transparency.

There are many ways that authors might employ generative AI: to summarize literature, formulate ideas, organize outlines, produce drafts of text, or revise and refine text (Gordijn and Ten Have 2023; Lund et al. 2023; Teixeira and Tsigaris 2023). Some possible uses do not seem significantly different from using internet searches, autocorrect tools, and grammar checks: authors might use generative AI to locate and understand scholarly material and draft text more efficiently. Other uses could influence content and style in novel ways. For example, authors might direct generative AI to propose questions that a paper might address, ideas that a paper might develop, possible outlines for a paper’s structure, or alternative phrasing for difficult or ambiguous passages. An author whose primary language is not English might employ generative AI to rewrite a draft and produce a more accessible final version. In these cases, generative AI would serve to produce prompts, suggestions, or foils for the authors’ thinking. Yet other uses could raise difficult and maybe novel questions about how ideas and text have been produced and whether they rightly belong to the person. Imagine, for example, that an author used generative AI to mimic the substance and style of another scholar. Questions about plagiarism would arise in such a case, even if no specific passages could be traced to the other scholar.

Authors who employ generative AI in developing papers should transparently disclose their use to editors, reviewers, and readers. Since generative AI is constantly changing and the scholarly community is only beginning to experiment with it, it is not prudent at this time to promulgate hard and fast rules for how generative AI should be disclosed. We recommend, however, that disclosure should describe how the AI was used and should identify AI-generated content. Authors should err on the side of too much transparency rather than too little: when in doubt, disclose. Some ways of disclosing the use of generative AI could include describing the use in a paper’s introduction, methods section, appendix, or supplemental material or citing the generative AI tool in the notes or references.

Although editors must rely on authors to honestly and transparently disclose their use of generative AI, editors should have access (through their publishers or through other services) to tools that can detect whether generative AI was used (and potentially how it was used) in a submitted paper. As with tools that are employed to check for plagiarism, detection tools for generative AI are unlikely to be foolproof. Therefore, the ability of editors to continually draw upon a community of expert reviewers who can raise concerns about an author’s use of generative AI will also be essential.

Fully transparent disclosure is important for several reasons:

*To flag potential problems regarding the accuracy of information.* For the time being, generative AI is extremely unreliable at offering accurate citations and often makes factual and reasoning errors. In the future, new versions of generative AI and add-ons may be more reliable, but existing systems are, as the name ChatGPT implies, generative transformers of information more than reliable reporters. Authors, readers, and reviewers need to be alerted to, and on guard against, the possible presence of erroneous information.

*To understand the origin of potential bias within ideas.* Generative AI tools may prove to be useful for helping authors collect, organize, and articulate their thoughts. When so used, the technology appears to be analogous to the online platforms and software that gather and analyze data for empirical research reports. Professional scholarly norms dictate that information about these tools be provided to help readers and reviewers evaluate a report. For example, it is common in survey research to cite the use of Qualtrics or REDCap for survey distribution as well as to specify uses of MTurk or specific panels used for survey recruitment. Software such as SPSS, Stata, or Prism are similarly cited when they are used as data analysis tools. Similarly, both for an empirical research report and for a paper that presents conceptual, philosophical analysis, explaining how generative AI has been used to help generate the paper might be necessary to help readers and reviewers evaluate it.

*To assess ownership and protect the community of scholars.* Just as AI image generators can be trained on a visual artist’s work and asked to create images in that artist’s style, large language models can be trained (so-called fine-tuned) with a writer’s work and asked to generate text that mimics that writer, stylistically and substantively. In some cases, such uses will raise questions about plagiarism or intellectual property and about protecting the scholarly community. Creating a dialogue in the style of Plato’s Gorgias might be a creative and illuminating exercise for teaching, but generating a paper by training a large language model on the work and style of a living author could harm that author and undermine the community’s trust.

*To support public deliberation about the uptake of generative AI.* There are calls from many sources, including from some AI developers, for a broad public conversation about the design and public oversight of these tools, given their implications for the accuracy of shared information and the construction of ideas and considering their potential risks for professional communities. Whatever form such a broad public conversation might take, it depends on a high level of public transparency about the use of generative AI.

### Editors and reviewers should not rely solely on generative AI to review submitted papers

Any uses editors make of generative AI should also be transparent to authors and should not be the sole basis of reviewer recommendations or editorial decisions. One rationale for this proscription is again to safeguard the editors’ role in fostering a community of scholars who are in extended conversation with each other and together sustain and grow their own community of experts. For the time being, given the current state of development of generative AI tools, we do not believe that they are adequate as reviewers (see also National Institutes of Health 2023).

However, just as with the creation of content, in the evaluation of a paper, generative AI might be used in a variety of ways; entirely replacing reviewers with AI is only the limit case. An editor might also ask an AI tool whether the concepts or arguments presented in an article have ever appeared in published material. Using AI in this way is similar to running a paper through plagiarism-detection software to determine whether blocks of text have previously been published, even though the use would be intended to gauge conceptual novelty, not to detect actual plagiarism.

In light of the potential for improved efficiency and timeliness, there is likely to be pressure by publishers to rely increasingly on AI as a substitute for peer reviewers. Despite the many challenges and difficulties with peer review, we believe that a complete substitution should not take place and urge that publishers retain humans as the final arbiters in the review process.

### Editors retain final responsibility in selecting reviewers and should exercise active oversight of that task

It is also possible that generative AI could be used to identify peer reviewers for manuscripts. Given that many editors already rely on software to suggest peer reviewers and on algorithms to remove conflicts of interest and check for publications in the relevant areas, it would be unsurprising for AI to play a growing role in the editorial process. Again, using AI as a decision-support tool may be beneficial and save time. But replacing this editorial function with AI seems unwarranted, except under exceptional circumstances. There are advantages to having a person—an agent who has moral responsibility for the content of a journal—standing behind all editorial decisions. This has the potential to be lost in new publication models that have moved away from having an editor-in-chief role. It remains to be seen how a sense of editorial responsibility will be distributed in these new publishing models, though ethics audits and greater responsibility by the publisher combined with advisory boards of scholars have been explored.

### Final responsibility for the editing of a paper lies with human authors and editors

In principle, copyeditors could employ generative AI to improve the language and style of a manuscript and to bring it into conformity with internal guidelines for formatting and references. Such uses do not appear to be substantively different from authors’ uses of AI to revise and refine a manuscript in the final stages of preparation, prior to submission. In keeping with the positions above, final responsibility for the text must lie with humans, however.

## Toward shared norms

The stance set out here is consistent with those taken by the Committee on Publishing Ethics and many journal publishers, including those that publish or provide publishing services to the journals we edit. Previous position statements have addressed concerns about the use of AI for peer review and the importance of reviewers revealing to authors if they used AI in their review (Zielinski et al. 2023). However, to our knowledge, none have addressed the importance of using human reviewers to review manuscripts and editors retaining final decisions over what reviewers to select. Our stance differs from the position of *Science* magazine, which holds not only that a generative AI tool cannot be an author but also that “text generated by ChatGPT (or any other AI tools) cannot be used in the work, nor can figures, images, or graphics be the products of such tools” (Thorpe 2023, p. 313). Such a proscription is too broad and may be impossible to enforce, in our view. Yet we recognize that the ethical issues raised by generative AI are complex, and we have struggled to decide how editors should promote responsible use of these technologies. Over time, we hope, the community of scholars will develop professional norms about the appropriate ways of using these new tools. Reviewers and readers, not just editors, will have much to say about these norms. The variety of ways in which generative AI technologies can be used and the pace of change may, in fact, render detailed editorial policy statements ineffective or impracticable. Instead, reliance on evolving professional norms based on broader public conversation about generative AI technologies may turn out to be the best way forward. Our shared statement is intended to promote this wider social discourse.

## Declarations

### Authors and Signatories

*Gregory E. Kaebnick* is the editor of the *Hastings Center Report*; *David Christopher Magnus* is the editor in chief of the *American Journal of Bioethics*; *Audiey Kao* is the editor in chief of the *AMA Journal of Ethics*; *Mohammad Hosseini* and *David Resnik* are associate editors for *Accountability in Research*; *Veljko Dubljevic* is the editor in chief of the *American Journal of Bioethics—Neuroscience*; *Christy Rentmeester* is the managing editor of the *AMA Journal of Ethics*; *Bert Gordijn* is a co-editor in chief of *Medicine, Health Care and Philosophy*; *Mark J. Cherry* is the editor of the *Journal of Medicine and Philosophy*; *Karen J. Maschke* is the editor of *Ethics & Human Research*; *Lisa M. Rasmussen* is the editor in chief of *Accountability in Research*; *Laura Haupt* is the managing editor of the *Hastings Center Report* and *Ethics & Human Research*; *Udo Schüklenk* is a joint editor in chief of *Bioethics* and of *Developing World Bioethics*; *Ruth Chadwick* is a joint editor in chief of *Bioethics* and the commissioning editor for ethics of the *British Medical Bulletin*; and *Debora Diniz* is the joint editor in chief of *Developing World Bioethics*.
