# Correction: Editors’ statement on the responsible use of generative AI technologies in scholarly journal publishing

**DOI:** 10.1007/s11019-023-10183-7

**Published:** 2023-12-08

**Authors:** Gregory E. Kaebnick, David Christopher Magnus, Audiey Kao, Mohammad Hosseini, David Resnik, Veljko Dubljević, Christy Rentmeester, Bert Gordijn, Mark J. Cherry

**Affiliations:** 1 The Hastings Center, Putnam Country, USA; 2 Stanford University, Stanford, USA; 3 American Medical Association, Chicago, USA; 4 Northwestern University, Evanston, USA; 5 U.S. National Institute of Environmental Health Sciences, Durham, USA; 6 North Carolina State University, Raleigh, USA; 7 Dublin City University, Dublin, Ireland; 8 St. Edwards University, Austin, USA

**Correction: Medicine, Health Care and Philosophy**

10.1007/s11019-023-10176-6

In the original publication of the article, under the section “Toward shared norms” the following sentence “However, to our knowledge, previous position statements have not addressed the responsibilities of reviewers to authors (Zielinski et al. 2023)” was published incorrectly.

The correct sentence should read as follows “Previous position statements have addressed concerns about the use of AI for peer review and the importance of reviewers revealing to authors if they used AI in their review (Zielinski et al. 2023). However, to our knowledge, none have addressed the importance of using human reviewers to review manuscripts and editors retaining final decisions over what reviewers to select.”.

